# Cognitive Impairment and Delirium in Older Patients Undergoing Major Head and Neck Surgery

**DOI:** 10.1177/01945998211045293

**Published:** 2021-09-21

**Authors:** David P. Goldstein, Michael Blasco, John de Almeida, Jie Su, Wei Xu, Marc Cohen, Michael Sklar, Shabbir Alibhai

**Affiliations:** 1 Departments of Otolaryngology–Head and Neck Surgery and Surgical Oncology, Margaret Cancer Centre, University Health Network, University of Toronto, Toronto, Canada; 2 Department of Biostatistics, Margaret Cancer Centre, University Health Network, University of Toronto, Toronto, Canada; 3 Head and Neck Service, Memorial Sloan Kettering Cancer Center, New York, New York, USA; 4 Interdepartmental Division of Critical Care Medicine, University of Toronto, Toronto, Canada; 5 Department of Medicine, University Health Network, University of Toronto, Toronto, Canada

**Keywords:** cognitive impairment, delirium, older patients, head neck surgery, clock draw test

## Abstract

The study objective was to measure the prevalence and predictors of cognitive impairment (CI) and delirium. Adults undergoing major head and neck cancer surgery completed the Clock Draw Test to screen for CI, defined as a score of 0 or 1. Postoperative delirium was recorded. Predictors of delirium and length of stay were assessed by univariate logistic regression and the latter with multivariate linear regression. Overall 274 patients were included, of which 47% had a Clock Draw Test score of 0 or 1. Post-operative delirium occurred in 17 (6%). CI was a predictor of postoperative delirium (odds ratio, 3.9; 95% CI, 1.2-12; *P* = .02). Postoperative delirium was a predictor of increased length of stay (adjusted odds ratio, 1.30; 95% CI, 1.07-1.57; *P* = .0073) on multivariate regression while baseline Clock Draw Test result was not a predictor on univariate regression (*P* = .98). Screening for CI can help predict delirium and facilitate targeted interventions in the postoperative period.

There is growing interest in the impact of geriatric syndromes on outcomes following head and neck cancer (HNC) treatment. In a letter to the editor, Parham highlights the importance of evaluating cognitive function and delirium in patients with HNC.^1^ Postoperative delirium has been reported with varying frequency following HNC surgery,^2^ with prior cognitive impairment (CI) being a risk factor.^3^ Studies have been primarily retrospective, with CI being determined on medical history.^2,4^ A number of simple and easy-to-use screening tests have been devised to help identify patients with CI. One such test is the Clock Draw Test (CDT), which requires visuospatial orientation, concentration, and planning ability. The CDT has been demonstrated to be able to detect mild CI as well as dementia.^5,6^ The objective of this report was to present results of the prevalence and predictors of CI and delirium in a previously reported prospective study on frailty in older patients undergoing major HNC surgery.^7^

## Methods

A prospective study of patients aged ≥50 years undergoing major HNC surgery was performed. University Health Network research ethics board approval was obtained, and patients provided written informed consent for participation. Eligible patients at baseline completed the CDT to screen for CI, with Fried’s frailty score and the VES-13 (Vulnerable Elders Survey). For the CDT, patients were asked to draw the numbers of a clock and the hands of an assigned time; this was evaluated using similar criteria to the Montreal Cognitive Assessment, where each task is scored incorrect (0) or correct (1) and then summed for a total of 0, 1, or 2.^8^ Patients were considered to have CI if they had a CDT score of 0 or 1. Postoperative outcomes were collected, including complications and length of stay (LOS). Postoperative delirium was recorded per the clinical team’s assessment of the presence of restlessness, agitation, hallucinations, or altered level of consciousness. The proportion of patients with preoperative CI and postoperative delirium was determined, and differences were evaluated by chi-square analysis. Predictors of delirium were assessed by univariate logistic regression. To evaluate predictors of LOS, log transformation was performed, as LOS was a nonzero right-skewed outcome, and univariate regression was applied. Multivariate linear regression was performed via a backward selection algorithm. Odd ratios (ORs) and regression coefficients (β) for LOS were calculated with 95% CIs.

## Results

An overall 274 patients were enrolled. At baseline, 22 patients (8%) had a CDT score of 0; 108 (39%), a score of 1; and 144 (53%), a score of 2. Postoperative delirium occurred in 17 patients (6%). **Table 1** highlights the variables associated with CDT scores. On univariate regression, predictors of postoperative delirium included CI (OR, 3.9; 95% CI, 1.2-12; *P* = .02) and a higher frailty score (OR, 1.57; 95% CI, 1.09-2.25; *P* = .015). Age (OR, 1.05; 95% CI, 0.996-1.11; *P* = .075) and VES-13 score (OR, 1.24; 95% CI, 0.96-1.61; *P* = .1) were not associated with development of postoperative delirium. On univariate regression, postoperative delirium was a predictor of increased LOS (β = 1.48; 95% CI, 1.07-1.95; *P* = .016), while baseline CDT was not (*P* = .98). On multivariate regression (**Table 2**), delirium remained an independent predictor of increased LOS (β = 1.30; 95% CI, 1.07-1.57; *P* = .0073). Thus, the LOS was 30% longer for patients with delirium than those without. CI was not a predictor of grade of complication (*P* = .76), medical complications (*P* = .1), or surgical complications (*P* = .11).

**Table 1.** Variables Associated With Clock Draw Test Scores on Chi-square Test.

		Clock Draw Test score			
Covariate	Full sample	Abnormal: 0	Borderline: 1	Normal: 2	*P* value
No.	274	22	108	144	
Frailty					.0047
No frailty	119 (43)	5 (23)	43 (40)	71 (49)	
Prefrailty	132 (48)	11 (50)	54 (50)	67 (47)	
Frailty	23 (8)	6 (27)	11 (10)	6 (4)	
VES-13					.03
Mean (SD)	2.9 (1.6)	3.7 (2)	3.3 (2)	2.7 (1.2)	
Median (min-max)	2 (0-9)	3 (2-7)	3 (0-9)	2 (2-8)	
Missing	38	9	17	12	
Age, y					<.001
50-64	105 (38)	4 (18)	27 (25)	74 (51)	
≥65	169 (62)	18 (82)	81 (75)	70 (49)	

Abbreviation: VES-13, Vulnerable Elders Survey–13.

a Values are presented as No. (%) unless noted otherwise.

**Table 2.** Multivariable Analysis for Predictors of Length of Stay.

Covariate	Estimate (95% CI)	Global *P* value^a^
Delirium		.0073
0	Reference	
1	1.30 (1.07-1.57)	
Tumor site		<.001
Skin, thyroid, salivary gland	Reference	
Oral cavity, oropharynx, hypopharynx, larynx	1.99 (1.80-2.23)	
Comorbidity score		.0034
Mild, none	Reference	
Moderate, severe	1.16 (1.05-1.27)	
Free flap		.003
No	Reference	
Yes	1.31 (1.08-1.46)	
Operative time per hour	1.06 (1.03-1.08)	<.001
Preoperative hemoglobin per 10-unit change	0.96 (0.93-0.99)	.0069

## Discussion

Using the CDT as a screening measure for CI, we demonstrated that just under half the sample screened positive. Had CI been based on a history of dementia, we would have identified only 1 patient who was reported to have mild dementia per the comorbidity index. Older, frail, and vulnerable patients were found to be at increased risk of CI, which has been shown in other studies.^2,4^ Patients with risk factors and those who screen positive for CI preoperatively would likely benefit from a more comprehensive preoperative assessment to determine extent of preimpairment.

Postoperative delirium was reported in only 6% of our patients, which is lower than what is reported in the literature, in which delirium incidence ranges from 10.9% to 36%.^2,4^ The lower rate of delirium in our series may be related to the fact that a standardized tool was not used to prospectively measure and record delirium; thus, the prevalence may be underreported. We found that CI, as measured by the CDT, was associated with postoperative delirium with increasing frailty status, while chronologic age was not. Although larger studies are needed to validate these findings given our low number of patients with delirium, approaches to early identification of those at risk of postoperative delirium would include simple CI measures as well as frailty measures. Chronologic age in of itself is not a useful predictor, as shown in our study.

Delirium was an independent predictor of increased LOS. Predicting delirium preoperatively may facilitate targeted interventions in the postoperative period to prevent delirium and decrease LOS. Humeidan et al recently reported in a randomized trial that patients who underwent and were at least minimally compliant with cognitive prehabilitation (cognitive exercise targeting memory, speed, attention, flexibility, and problem-solving functions) had a lower postoperative delirium risk.^9^ We feel that it is important to further evaluate cognitive function and delirium, as well as pre- and postoperative interventions in patients undergoing HNC surgery.
